# Gut Microbiota Dysbiosis and Its Role in the Development of Irritable Bowel Syndrome

**DOI:** 10.7759/cureus.83084

**Published:** 2025-04-27

**Authors:** Manal M Saleem, Sarah Masood, Maryam M Rahmatullah, Ifrah Ayesha Imdad, Asma Mohammed Aslam Sange, Dina Nasr

**Affiliations:** 1 College of Medicine, Dubai Medical University, Dubai, ARE

**Keywords:** dysbiosis, fecal transplantation, irritable bowel syndrome, microbiome, probiotics

## Abstract

The gut microbiota refers to the diverse community of symbiotic and pathogenic microorganisms inhabiting the host digestive tract. This microbiome plays a vital role in maintaining the integrity of the digestive system. Irritable bowel syndrome (IBS) is a functional gastrointestinal disorder (FGID) characterized by chronic abdominal pain and altered bowel habits. Although the pathophysiology of IBS remains unclear, recent studies suggest that the disruption of the gut microbiota (dysbiosis) may play a significant role. This study aims to examine the role of the gut microbiota in the development of IBS, analyze factors influencing the gut microbiome, and explore the potential for microbiota-targeted therapies. Relevant literature published from 2014 until 2024 was sourced from Google Scholar, PubMed, and Scopus using the keywords "microbiome", "irritable bowel syndrome", "dysbiosis", "faecal transplantation", and "probiotics". This review revealed consistent evidence of gut microbiota dysbiosis in individuals with IBS, characterized by altered microbial diversity, composition, and metabolic function. Contributing factors included a reduced abundance of beneficial commensals, overgrowth of potentially pathogenic species, and disrupted host-microbiota interactions. This dysbiosis was also frequently associated with symptom severity and specific IBS subtypes. Emerging evidence further highlights the role of diet, stress, and genetic factors in modulating gut microbiota and influencing IBS development. The growing body of research supports a strong link between dysbiosis and the pathogenesis and symptomatology of IBS. Understanding the microbial underpinnings of IBS opens avenues for potential diagnostic biomarkers and innovative therapeutic interventions aimed at restoring a balanced gut microbiota. However, further research is needed to elucidate the underlying mechanisms and translate these insights into effective clinical strategies for the management of IBS. This review underscores the significance of gut microbiota in IBS and its potential as a target for future therapeutic interventions.

## Introduction and background

Introduction to irritable bowel syndrome (IBS) 

Irritable bowel syndrome (IBS) is a highly prevalent functional gastrointestinal disorder (FGID), now classified under disorders of gut-brain interaction (DGBIs). It is characterized by recurrent abdominal pain associated with altered bowel habits, including diarrhea, constipation, or a combination of both [1]. Symptom intensity varies widely among patients, ranging from tolerable to severe, with some experiencing daily symptoms and others only at intervals of weeks or months [2].  

Pathophysiology of irritable bowel syndrome (IBS) 

Only a portion of the pathophysiological pathways underlying IBS are currently understood. Various patient subgroups have been identified, exhibiting abnormal gastrointestinal (GI) motility, visceral hypersensitivity, altered brain-gut function, low-grade inflammation, and psychosocial instability. Microenvironmental factors, such as gut microbiota, may overstimulate the mucosal immune system when the intestinal epithelial barrier is compromised. This immune activation leads to abnormal signaling through both extrinsic afferent and enteric nerves, potentially disrupting intestinal physiology and sensory perception, thus contributing to IBS symptoms [1]. 

Etiology 

IBS may also be triggered or exacerbated by genetic and environmental factors, such as dietary habits and prior infections. The identification of these variables and their interaction with the brain has ushered in a completely new era in the understanding, acknowledgment, and validation of IBS and other FGIDs. To date, one of the strongest risk factors for the development of IBS remains infectious gastroenteritis [3]. Recently, increased attention has been given to the impact of genetic factors, particularly the substantial genetic overlap between IBS and mental health disorders such as depression and anxiety. This connection suggests that the same brain-gut pathways implicated in mood disorders may also influence IBS, highlighting the potential for psychobiotic treatments and other comprehensive strategies targeting both GI and psychological symptoms [4]. 

Epidemiology 

According to a recent review, IBS affects approximately 11% of the global population, with the highest prevalence (21%) observed in South America and the lowest (7%) in South Asia [5]. However, due to variations in diagnostic criteria and survey methodologies across studies, accurately determining prevalence remains challenging [5-7].  

Diagnostic criteria 

IBS is classified into several subtypes: IBS with constipation (IBS-C), IBS with diarrhea (IBS-D), mixed IBS (IBS-M), and unclassified IBS (IBS-U) [7]. The Rome IV criteria are currently the most widely used for diagnosing IBS [3]. According to these criteria, individuals must experience recurrent abdominal pain at least one day per week over the past three months, associated with at least two of the following factors: improvement or worsening with defecation, changes in stool frequency (such as constipation, diarrhea, or both), or changes in stool form or appearance. Additionally, symptoms must have been present for at least six months prior to diagnosis [6,8].  

Health-related quality of life (HRQOL) and irritable bowel syndrome (IBS) 

IBS has been shown to significantly reduce social engagement, decrease workplace productivity, and limit everyday activities, all of which adversely impact HRQOL. Patients' overall well-being is further compromised by increased fatigue, heightened health concerns, and role limitations [9]. These impairments are largely due to several comorbidities that substantially affect the quality of life of individuals with IBS. Between 40% and 60% of IBS patients also experience mental health conditions, such as depression, panic disorder, and generalized anxiety disorder (GAD). These conditions may amplify GI symptoms, initiating a vicious cycle in which mental health problems worsen GI problems and vice versa [10,11]. The gut-brain axis (GBA) plays a critical role in this relationship, as gut motility and sensitivity are influenced by psychological factors. Functional MRI studies have demonstrated increased sensitivity in brain regions associated with pain perception among IBS patients, highlighting the connection between GI function and psychological distress [11]. Furthermore, fibromyalgia and other chronic pain syndromes are common in IBS patients, increasing both symptom burden and healthcare utilization [12]. Poor sleep quality, also prevalent among individuals with IBS, has been shown to exacerbate symptoms and contribute to functional impairment [13]. Additionally, a significant proportion of IBS patients suffer from GI disorders, such as inflammatory bowel disease (IBD) and gastroesophageal reflux disease (GERD). These findings underscore the multifactorial nature of IBS and the importance of considering both biological and psychological aspects in its management.

Gut microbiota 

The human gut microbiota consists of trillions of microorganisms, including bacteria, viruses, and fungi, which play crucial roles in maintaining host health. A balanced gut microbiome is essential for the normal development and function of the intestinal immune system and also supports general GI function, barrier integrity, and modulation of permeability [14]. The gut microbiota is involved in numerous physiological processes, including digestion, immune system regulation, nutrient synthesis, and the maintenance of GI homeostasis. Characterizing the microbiome in healthy individuals is a critical first step in understanding how the microbiome contributes to both health and disease. A healthy human adult gut typically harbors approximately 1,000 different bacterial species, with Firmicutes and *Bacteroides *recognized as two dominant phyla [15]. Colonization of the gut microbiota begins immediately after birth and continues until maturity. Early microbial composition varies based on delivery mode and feeding method, with vaginally delivered and cesarean-delivered infants showing distinct microbial profiles. Additional influences include the mother's microbiota during pregnancy and whether the infant is formula-fed or breastfed. By around three years of age, children typically develop a gut microbiota composition resembling that of adults (i.e., a pattern abundant in both quantity and diversity). However, before it reaches a more stable state, it goes through several substantial modifications and transitions during childhood [16].

Composition of the gut microbiome 

The normal healthy gut microbiome is composed of nearly 1,000 different bacterial species, primarily belonging to six phyla: Firmicutes (64%), Bacteroidetes (23%), Proteobacteria (8%), Actinobacteria (3%), Fusobacteria, and Verrucomicrobia [16]. A common finding in dysbiosis is a decrease in butyrate-producing microbes and those associated with lower luminal lipopolysaccharide (LPS) levels, improved intestinal barrier function, and decreased visceral fat mass. These beneficial microbes include the families Rikenellaceae and Christensenellaceae; the genera *Bifidobacterium*, *Oscillospira*, and *Akkermansia*; and the species *Alistipes finegoldii*, *Alistipes senegalensis*, and *Faecalibacterium prausnitzii*). Dysbiosis is also characterized by an increase in opportunistic pathogens, including hydrogen producers and LPS-producing bacteria. Gram-positive Firmicutes have been proposed to extract calories more efficiently from carbohydrates compared to gram-negative Bacteroidetes. Firmicutes increase the fermentation of otherwise indigestible carbohydrates into small-chain fatty acids, which are subsequently absorbed and utilized for gluconeogenesis and lipogenesis. An increased Firmicutes-to-Bacteroidetes (F:B) ratio has been widely studied as a potential biomarker for metabolic diseases and obesity and has been found to be disrupted in patients with IBS. Additionally, certain bacteria, such as *Escherichia coli*, *Bacteroides* species, and chiefly *Prevotella* species, can damage the intestinal barrier, whereas others, like *Akkermansia muciniphila* and *F. prausnitzii*, help preserve its integrity. Bacteria associated with metabolic diseases, such as *Ruminococcus*, *Fusobacterium*, and *Staphylococcus aureus*, are frequently implicated in promoting inflammation [16].

Gut microbiota and disease 

Recent epidemiological and physiological research, supported by cellular and animal studies, has shown that the intestinal microbiota plays a crucial role in influencing both health and disease. Rather than the presence of a specific strain or species, the variability and diversity of the gut microbiome are believed to play a major role in the development of these disorders. It is the interaction and diversity of species working in symbiosis that needs to be taken into account when investigating the biochemical and metabolic processes of the disease [16,17]. 

Dysbiosis 

Dysbiosis refers to a disruption or imbalance in the composition of the gut microbiota and will be further elaborated upon throughout this review [17]. Dysbiosis is an imbalance in microbial communities that can negatively impact the gut-organ axis, leading to various health issues across multiple organ systems. Dysbiosis has been implicated in the development of various diseases, including IBS, which is due to an increased abundance of Firmicutes and a reduction in Bacteroidetes. Additionally, the gut microbiota and its metabolites are linked to numerous health conditions, such as non-alcoholic fatty liver disease (NAFLD), IBD, liver cancer, cardiovascular disease (CVD), alcoholic liver disease (ALD), chronic kidney disease (CKD), and cirrhosis [18]. Recent studies have emphasized the importance of gut microbial homeostasis, known as eubiosis, which represents an optimal balance that supports health and well-being. Maintaining eubiosis, alongside appropriate dietary intake, has been shown to benefit human health and help prevent or manage microbial-related diseases. Imbalances in the gut microbiota, often driven by excessive antibiotic use, are increasingly recognized as contributors to systemic illnesses [19].  Host factors such as age, diet, lifestyle, and environmental exposures significantly influence gut microbial diversity, with diet currently considered one of the most critical modulators [20]. Thus, the interaction between host and microbiota plays a pivotal role in health and disease.

## Review

Dysbiosis and the onset of irritable bowel syndrome (IBS) 

There is a complex relationship between the gut microbiome and the development of IBS. The gut microbiota plays a crucial role in maintaining intestinal barrier function, nutrient absorption, and immune responses. Disruption of microbial balance can increase gut permeability (often referred to as a "leaky gut"), allowing the translocation of bacteria and bacterial products that trigger immune responses and cause inflammation, processes linked to the onset and progression of IBS. IBS patients often exhibit altered gut microbiomes, characterized by lower levels of beneficial bacteria, such as *F. prausnitzii* and *Bifidobacterium*, and higher levels of potentially harmful bacteria like Enterobacteriaceae [1]. A meta-analysis of 23 studies involving 1340 participants revealed that, compared to healthy controls, IBS patients had greater amounts of *E. coli* and *Enterobacter *and lower levels of *Lactobacillus *and *Bifidobacterium *[21]. Patients have also shown altered levels of microbial metabolites, such as short-chain fatty acids (SCFAs) and bile acids, suggesting that microbial by-products may contribute to IBS symptoms [22]. In addition, one study found that microbial imbalances can impair the GBA, resulting in visceral hypersensitivity and altered GI functioning, thereby triggering IBS symptoms [23]. 

Dysbiosis and the severity of irritable bowel syndrome (IBS) 

IBS symptoms, such as pain, bloating, and altered bowel habits, are associated with dysbiosis, which increases intestinal permeability, activates the immune system, and causes low-grade inflammation [24]. Growing evidence suggests that dysbiosis is a key contributor to IBS [21]. A study by Tap et al. [25] identified specific gut microbiota signatures associated with IBS severity, demonstrating that patients with severe IBS symptoms had distinct microbial compositions compared to those with milder symptoms and healthy controls [25]. Notably, severe IBS was linked to a reduction in beneficial bacterial species, such as *Faecalibacterium *and *Bifidobacterium*, and an increase in potentially pathogenic bacteria. These findings suggest that dysbiosis not only contributes to the presence of IBS but may also influence symptom severity, providing potential targets for microbiota-based interventions.  Continued research into these microbial signatures could help refine diagnostic criteria and therapeutic options tailored to the microbiome profiles of different IBS subtypes. Developing predictive models using microbial markers may guide personalized therapies (e.g., specific probiotics, dietary interventions, or microbiota-directed drugs).

Environmental influences on gut microbiota  

A healthy gut microbiota is shaped largely by environmental influences, which play a crucial role in maintaining gut health. Several factors can disrupt the balance of gut bacteria, including diet, antibiotic use, and lifestyle choices. Among these, diet is currently considered one of the most significant modulators of the gut microbiota [20].  

Diet and medication use 

Fermentable oligosaccharides, disaccharides, monosaccharides, and polyols (FODMAPs), especially fermentable carbohydrates, can negatively alter the gut microbiota when consumed alongside diets high in processed foods and low in fiber. Changes in microbial diversity and metabolic activity associated with such dietary patterns are linked to dysbiosis and the development of IBS. As a result, dietary and lifestyle modifications are crucial for maintaining gut health and preventing IBS. This highlights the significant influence of diet on gut health and disease management [14]. Personalized dietary treatments also hold promise. A recent clinical trial compared a low-FODMAP diet and traditional dietary advice with a low-carbohydrate diet and pharmacological treatment, demonstrating that specific diets were more effective for specific IBS subgroups, thus supporting customized dietary approaches [4,26]. Moreover, research linking variations in carbohydrate-processing genes (CAZymes) to dietary response suggests that genetic profiling could enhance dietary recommendations, allowing for more precise matching of dietary interventions to patients' genetic profiles [26]. A study involving 10 healthy participants, aged 21-33, who alternated between plant-based and animal-based meals over five consecutive days showed that the animal-based diet increased bile-tolerant bacteria and decreased fiber-fermenting bacteria [16]. Similarly, in mice, high-fat diets influenced microbial ecosystems, including an increased abundance of monounsaturated Bacteroidaceae and *Bifidobacterium *with ω-3 polyunsaturated fatty acids and a decreased abundance of *Bacteroides*. Dietary saturated fats were associated with obesity and multiple sclerosis (MS). According to the CORDIOPREV study, persons with obesity and/or MS may benefit from both Mediterranean and low-fat diets in improving dysbiosis [16]. Probiotic and prebiotic treatments are also emerging as promising strategies. Probiotics have shown potential in managing IBS symptoms by restoring gut flora balance and reducing GI issues, as highlighted in multiple reviews and meta-analyses [23,27]. 

Antibiotic use and other medications that disrupt microbial balance can also negatively affect individuals susceptible to gut dysfunction. It has been established that antibiotics can significantly alter gut microbiota by reducing microbial diversity and shifting bacterial composition. Researchers emphasize that antibiotics not only eliminate pathogenic bacteria but also damage beneficial species, leading to dysbiosis. This disruption can promote the overgrowth of harmful bacteria, contributing to GI and other health issues. A study published by Modi et al. highlighted the potentially harmful effects of antibiotics on the gut microbiota, with effects persisting for months or even years [28]. Understanding these effects is critical to developing strategies that minimize antibiotic-associated dysbiosis [28]. Antibiotics disrupt pathogenic and commensal gut microbiome composition, with the majority of adverse effects seen in prenatal and early-life exposure to antibiotics. A randomized controlled trial by Wei et al. studied children aged 12-36 months exposed to azithromycin, a macrolide antibiotic, was used to show the effects of antibiotics on gut microbiota, and found that just 14 days of treatment significantly decreased microbiota diversity and richness, particularly affecting the Actinobacteria phylum and reducing the abundance of *Bifidobacterium *[29]. Furthermore, other medications such as proton pump inhibitors and antineoplastics were also shown to alter the gut microbiome [16]. Moreover, lifestyle interventions focusing on dietary habits, stress management, and sleep hygiene are critical strategies for maintaining a balanced gut microbiota and preventing GI issues such as IBS.

Mental well-being and gut microbiota 

Stress and physical activity are pivotal environmental factors influencing the gut microbiota. Chronic stress has been shown to decrease populations of beneficial bacteria, disrupt the GBA, alter microbial diversity, and promote the growth of harmful bacteria. Individuals with GI disorders such as IBS may experience symptom exacerbation due to elevated levels of stress hormones, which impair gut integrity, promote inflammation, and foster dysbiosis. Similarly, inconsistent sleep habits negatively affect gut health by disrupting circadian rhythms, which are important for the regulation of gut motility and immunity. In contrast, regular physical activity promotes gut health by reducing systemic inflammation, increasing beneficial microbial species, and enhancing microbial diversity. Thus, sustaining a healthy gut microbiome through stress reduction, sufficient sleep, and regular exercise may help prevent GI issues such as IBS [30].  Additionally, emerging interventions such as probiotics and fecal microbiota transplantation (FMT) offer promising avenues for restoring gut health following disturbances [28]. 

Probiotics and their mechanism of action 

Probiotics are live microorganisms that, when consumed in adequate amounts, provide health benefits by supporting the gut and overall health. Their mechanisms of action include enhancing the intestinal barrier, modulating immune responses, producing antimicrobial substances, and competing with pathogens for adhesion sites. By improving gut microbiota composition, probiotics help maintain GI balance, reduce inflammation, and protect against harmful bacteria, making them a promising therapeutic option for conditions like IBS [21]. 

A study suggests that probiotics can modulate the gut microbiota, reduce visceral hypersensitivity, and exert anti-inflammatory effects in IBS patients. Several mechanisms suggest how probiotics might benefit IBS patients. First, many probiotic strains, such as *Lactobacillus* and *Bifidobacterium*, demonstrate antibacterial and antiviral properties that could prevent or modify the course of post-infective IBS. Second, probiotics reduce mucosal inflammation, thereby decreasing immune-mediated activation of enteric motor and sensory neurons and modulating neural traffic between the gut and central nervous system (CNS). Third, probiotics can alter the composition of the gut flora. Finally, probiotics may influence stool and gas composition or enhance intestinal mucus secretion, thereby improving symptoms such as constipation and diarrhea. However, the effects of probiotics can vary significantly depending on the strain, dosage, and duration of treatment, and they are not universally effective for all IBS patients [21].  

According to Didari et al., probiotics can alleviate IBS symptoms such as bloating, abdominal pain, and stool irregularities [23]. Their beneficial effects are attributed to restoring gut microbial balance, counteracting the dysbiosis often seen in IBS patients, which includes reduced microbial diversity and lower levels of beneficial bacteria like *Bifidobacterium *and *Lactobacillus*. Additionally, probiotics exhibit antimicrobial properties that protect against pathogenic bacteria, further supporting immune modulation and symptom relief [25]. 

In conclusion, probiotics offer a promising therapeutic approach for managing IBS by modulating gut microbiota and alleviating symptoms such as bloating and abdominal pain. Their effectiveness varies based on specific strains, dosage, and treatment duration, highlighting the need for personalized probiotic therapy. Key mechanisms include antibacterial properties, anti-inflammatory effects, and the restoration of beneficial gut flora, all contributing to improved quality of life for IBS patients. Although generally safe, caution is advised in individuals with underlying health conditions. Integrating probiotics into a comprehensive management plan, alongside dietary modifications and other therapies, may enhance their benefits. Continued research is crucial to identify optimal strains and formulations, ultimately improving patient care in IBS.  

Management of IBS using FMT is also under investigation. FMT shows promise in restoring gut microbiome diversity, although individual factors may influence its efficacy [30,31].  

Fecal microbiota transplantation (FMT) in irritable bowel syndrome (IBS) 

FMT is an emerging treatment method with the potential to affect the prognosis of prevalent chronic diseases like metabolic syndrome, cancers, and autoimmune diseases. While FMT is currently approved for the treatment of *Clostridioides difficile* infections, its application to other illnesses remains under investigation.  

FMT involves transferring fecal material from a healthy donor into the recipient's GI tract to restore microbial balance. First used in the fourth century in traditional Chinese medicine as a treatment for “quenching thirst” (ancient term for diabetes), Ge Hong, the infamous alchemist, used a treatment like fecal bacterial transplantation “Huanglong Soup” to treat food poisoning and diarrhea [32]. As of now, FMT has been used to treat recurrent *C. difficile* infections, but accumulating data coming from several clinical trials clearly indicate that numerous other conditions, including GI and liver disorders, cancer, inflammatory and infectious diseases, autoimmune disorders, brain disorders, obesity, and metabolic syndrome, may be treated with FMT [33]. 

Composition of fecal microbiota transplantation (FMT) 

The main components of FMT are the gut flora of humans and other species [32]. FMT involves transferring the entire, stable fecal microbial community from healthy donors to a recipient with a disease associated with altered microbiota. The goal is to restore microbial balance (dysbiosis) and alleviate the symptoms of the disease [33]. The success of FMT depends on the microbial diversity and composition of donor feces [32]. 

Fecal microbiota transplantation** **(FMT) for central nervous system (CNS) disorders 

Dysbiosis of the gut microbiota has been closely linked to the pathogenesis and progression of depression, positioning it as a novel target for therapeutic intervention [34]. In clinical practice, evidence of microbiota-GBA interactions comes from the association between dysbiosis and CNS disorders (i.e., autism, anxiety-depressive behaviors) and FGIDs [35]. Current research suggests that the pathogenesis of IBS is influenced by interactions between the GBA, immune system, and intestinal microbiome [36]. A key mechanism for potentially correcting dysbiosis and improving neuropsychiatric outcomes is the microbial GBA. A preclinical study by Zhang et al. revealed that FMT can enhance neurochemical levels and reduce inflammatory markers [37]. 

Fecal microbiota transplantation (FMT) methodology 

FMT is generally considered safe, though its efficacy varies depending on factors such as donor selection and microbiome compatibility [38]. As with all transplantation procedures, FMT donors are carefully screened to ensure they are healthy. The current screening process aims to exclude individuals with diseases related to intestinal microbes, blood disorders, or bacterial or parasitic infections that could be transmitted through FMT, while also meeting inclusion criteria [36]. Patients who agree to accept FMT can be administered bacterial flora transplantation through three main approaches: upper, middle, and lower digestive tract. 

For the upper digestive tract, the two main methods are oral microflora liquid and oral microflora capsules. Nasointestinal tubes, endoscopic biopsy holes, percutaneous endoscopic gastrostomy, jejunal catheterization, and endoscopic catheterization, such as transendoscopic enteral tubing (TET), are all part of the middle digestive tract approach. The lower GI pathway includes colonoscopy, colostomy, enema, and colonic pathway TET [32]. Colonoscopy enables the lesion to be directly observed; however, being an invasive examination, colonoscopy may cause damage to the colon and is costly. Although the use of an enema is less financially straining and invasive than the colonoscopic approach, its performance is less satisfactory owing to the limited range of intestinal sites that the enema can reach [39]. However, research on the effect of the administration route for FMT on clinical efficacy is limited, and a large number of clinical trials are required for verification. The optimal administration route may depend on the location of the lesion, the characteristics of the disease, and the general condition of the patient [40].  

Fecal microbiota transplantation (FMT) efficacy 

FMT has shown effectiveness in treating *C. difficile* infections, but its role in managing IBS remains debated. Research presents mixed results, with some studies indicating symptom improvement, while others report no significant benefits.  For example, a study by El-Salhy et al. demonstrated significant improvements in fatigue and the quality of life in IBS patients who received FMT, concluding that FMT is an effective treatment for patients with IBS [38]. However, the success of FMT largely depends on factors such as the selection of a well-defined donor with a normal diagnostic index and a favorable microbial signature. As the dosage of FMT increases, so too do the responses to treatment. However, according to Zhang et al., IBS patients had varied responses to FMT [37]. A study by Johnsen et al. found that while FMT did not alter the microbiome of IBS patients, it could considerably alleviate their symptoms [41]. On the other hand, a 2018 study found that after taking FMT capsules, the microbiomes of IBS patients and donors were quite similar, with the microbiomes of the receivers being greatly affected by the FMT capsules [41]. Furthermore, a randomized placebo-controlled study done by El-Salhy et al. demonstrated that some IBS patients maintained symptom improvement for up to three months post-FMT, although the impact on the quality of life impact remained inconclusive [38]. Conversely, a systemic review and meta-analysis conducted in Europe in 2023 suggested that FMT may not improve the quality of life for IBS patients and could even reduce it [31]. In conclusion, while FMT offers short-term benefits, as demonstrated in various trials, its long-term efficacy remains uncertain. This highlights the need for further research to establish its long-term effectiveness and to better understand the underlying mechanisms [42]. 

Fecal microbiota transplantation (FMT) or probiotics: which has greater therapeutic potential

Both modalities (probiotics and FMT) have shown promising therapeutic potential, with certain probiotics effectively reducing IBS symptoms and reducing inflammation and FMT altering the composition of the gut microbiota, leading to longer-lasting symptom relief. According to the review, FMT offers the advantage of transferring the entire gut microecology as a functional "organ," unlike probiotics, which may only transfer a few microbial species into the host gut. It is also extremely safe, as it does not cause rejection or an immune response, unlike other organ transplantation [32]. On the other hand, probiotics are less invasive, easy to incorporate into daily life, exert anti-inflammatory effects, and reduce post-infectious IBS. However, probiotics are limited by the number of bacterial strains, whereas FMT provides nearly all the bacterial species present in healthy donors’ feces [43].

Overall, while FMT shows potential, patient characteristics must be carefully considered before its application in IBS, and further data are needed to support standardized treatment approaches.  

Future directions 

Future therapeutic directions for IBS emphasize integrated approaches that combine physiological treatments with psychosocial interventions. A major goal is to refine diagnostics and personalized treatment based on symptom subtypes, genetic factors, and microbiome composition. Genome-wide studies have revealed shared genetic pathways between IBS and mood disorders, highlighting the potential for tailored psychobiotic treatments that address both GI and mental health conditions, such as anxiety and depression. Furthermore, psychological therapies like mindfulness and cognitive behavioral therapy (CBT) play a crucial role in alleviating stress-induced GI symptoms. When combined with dietary, pharmacological, and microbial-based therapies, these approaches suggest a holistic model of IBS care that targets both physical and psychological dimensions [44,45]. 

Future research should focus on integrating microbiome-targeted therapies, such as probiotics and FMT, alongside personalized dietary interventions based on genetic and microbiome insights to optimize treatment outcomes. A comprehensive care model that emphasizes interdisciplinary collaboration among healthcare professionals can further enhance IBS management by addressing both the physiological and psychological aspects of the disorder.

## Conclusions

This review concludes by highlighting the crucial role of microbiota-focused therapies and the intricate involvement of gut microbiota in the pathophysiology of IBS. Dysbiosis, an imbalance in the composition of gut microbes, plays a central role in IBS symptoms by disrupting immune function, gut motility, and the GBA. Therapies targeting the microbiota, such as probiotics, dietary changes, and FMT, show potential in alleviating IBS symptoms by restoring microbial equilibrium. However, the variation in IBS presentation and individual responses to treatment underscores the need for personalized therapeutic approaches. To improve outcomes for IBS patients, further research is needed to deepen our understanding of the mechanisms underlying dysbiosis, to identify IBS-specific microbiota signatures, and to develop targeted microbiome-based therapies.
